# Developing infectious disease surveillance systems

**DOI:** 10.1038/s41467-020-18798-7

**Published:** 2020-09-30

## Abstract

Lessons learnt from the current pandemic will be invaluable to tackle a potential second wave, however, gaps remain in our readiness to face future pandemics. At *Nature Communications* we wish to support further research providing insights into how national and international systems could be shaped for increased preparedness to both local or global epidemics.

From early functional characterization of the SARS-CoV-2 spike protein^1^ to the development of a neutralizing monoclonal antibody^2^, from the observation of air transmission of the virus in ferrets^3^ to a case report of likely prenatal transmission^4^, *Nature Communications* has championed research that sheds light on key aspects of SARS-CoV-2 infection and the associated disease. We hope that publishing these studies has helped to provide directions for future research.

“At *Nature Communications*, with its multidisciplinarity and its Open Access model, we intend to provide a forum to discuss possible approaches to key issues that the pandemic has exposed, such as the reorganization of healthcare, of the economy, of cities and infrastructures, and so on.”

We also want to provide researchers with a platform to present findings and tools that can aid policy design and implementation. We are particularly interested in studies that combine digitally acquired data and computational modelling. To develop an effective disease surveillance system ready to assess epidemic waves and able to implement policies to tackle them, both have to work in unison. Many research teams have diverted their activity to pandemic-related research, some delivering results in extraordinarily short time spans. However, a lack of data at the level of individual contact patterns, policy compliance, and other aspects of human behaviour has hindered larger-scale modelling efforts to reconstruct the dynamics of a highly infectious disease. Collecting and sharing such data, though, should take into consideration individual privacy.

To highlight digital and computational studies relevant to epidemiological surveillance and control, we have compiled a Collection that we will update regularly. We hope the Collection will be useful both to researchers and decision-makers looking for epidemiological information with policy implications. Moreover, a system which can respond effectively to a pandemic has to be integrated into a variety of societal components and as such we hope that research presented in this Collection will also suggest possible evolutions of our society for a better quality of life for all. At *Nature Communications*, with its multidisciplinarity and its Open Access model, we intend to provide a forum to discuss possible approaches to key issues that the pandemic has exposed, such as the reorganization of healthcare, of the economy, of cities and infrastructures, and so on.

To identify key research questions for future investigation, we are launching the Collection with a series of survey and discussion articles. We reached out to experts in diverse fields of expertise (human behaviour, computational epidemiology, public health and data security) and asked them to recount their experience in contributing to the COVID-19 response and to provide their vision on how a disease surveillance system should be shaped^5^. They recall that even though a global data reporting initiative was put in place quickly, there are still no protocols for standardized data collection and sharing at a global level. They highlight the need to change routine socio-demographic data collection to increase spatial and temporal granularity to build actionable models that can be used to deploy effective policies for containment. This calls for a much more technologically advanced data collection system and a more pervasive one. Of course, this raises the question of how we can preserve personal freedom values in our societies and this has been an intense topic for debate, for instance, for the deployment of digital contact tracing apps^6,7^, which the decentralized framework^8^ has not extinguished entirely^9^. This is a key issue that is addressed in a commissioned Perspective^10^ that elaborates on possible frameworks to use digitally collected data (i.e. mobile phone tracing data) for epidemiological surveillance. Anonymized Call Data Records (location of the cell tower a user connects to) provide some level of information but more granular and sensitive information obtained from phone GPS or Bluetooth services allow for a fine-grained analysis of human mobility behaviour and contact patterns, respectively. Nevertheless, concerns remain on the potential inequalities that arise from technology-based frameworks. Subpopulations at risk, such as the elderly, or others whose contribution to disease transmission is more difficult to assess (e.g. children) are generally undersampled in this kind of data. As digitally collected data becomes more and more pervasive for infectious disease surveillance, we fully recognise the challenges associated with ensuring data accessibility while safeguarding personal privacy of collected data and as an Open Access journal, we will continue to work with the scientific community to meet those challenges.

The more detailed information fed into models, the more mechanisms and dynamics they can reproduce. Being able to physically ascertain the mechanics of disease spread will in turn improve the robustness of epidemiological models. It will also improve their predictive power which incidentally remains largely untested in computational epidemiology research for COVID-19. Still, as computational models become more and more integrated into decision making, there is a need for decision-makers to assess the purpose and reliability of models for the specific task at hand. As discussed in our Q&A^5^, there is also currently confusion on how decision making interacts with model outputs and on the fact that the latter do not automatically define the former. On these topics, we present a Comment^11^ that provides guidelines for decision-makers with questions that need to be addressed to assess the appropriateness of given models for specific tasks.

We hope this content inspires research that can bridge the gap between digital and computational research and decision making. *Nature Communications’* editors recognise the importance of such research to foster science-based policymaking and encourage such submissions to the journal.
